# Heart Failure with Preserved Ejection Fraction: Can Device Therapy Be Our Option?

**DOI:** 10.7759/cureus.7323

**Published:** 2020-03-19

**Authors:** Talha Ahmed

**Affiliations:** 1 Internal Medicine, University of Maryland Medical Center, Baltimore, USA

**Keywords:** heart failure, heart failure with preserved ejection fraction, implantable cardioverter defibrillator, cardiac resynchronization therapy, heart failure with reduced ejection fraction

## Abstract

Heart failure with preserved ejection fraction (HFpEF) comprises half of the total heart failure (HF) population. It is a unique class of patients whose systolic heart function is preserved but have impaired diastolic function leading to symptoms typical of HF. In the era of 1980s and 1990s, ‘congestive heart failure’ was used to refer to all the HF patients. With a better understanding of pathophysiology of ‘diastolic HF’, the term ‘HFpEF’ got widespread acceptance in early 2000s. Despite the increasing awareness of pathophysiology and diagnostic modalities for this group of HF patients, it is unfortunate to say that the therapies that we can provide are limited when compared to their counterpart HF with reduced ejection fraction (HFrEF) group. This review will focus on the use of device therapy in patients with HFpEF, particularly implantable cardioverter defibrillator and cardiac resynchronization therapy.

## Introduction and background

The traditional medications that are part of guideline-directed medical treatment (GDMT) for heart failure with reduced ejection fraction (HFrEF) patients were investigated in different trials; however, to the dismay of the investigators, none of these trials yielded positive results showing mortality benefits. Heart failure with preserved ejection fraction (HFpEF) patients continue to present the long-term management challenge especially when it is related to reducing mortality. There is a consensus regarding the management of HFpEF patients, which basically involves aggressive modification of risk factors such as blood pressure control diabetes management, while using diuretics to maintain the volume status [1]. The pathophysiology of patients with HFpEF involves a state of microvascular damage with chronic inflammation. This indicates that these patients could benefit from device therapy (implantable cardioverter defibrillator or cardiac resynchronization therapy). However, there does not exist any established indication for such therapy in this patient population as opposed to HFrEF patients who have established indications for device therapy [2].

## Review

HFpEF comprises half of the total heart failure (HF) population. It is a unique class of patients whose systolic heart function is preserved but have impaired diastolic function leading to symptoms typical of HF. In the era of 1980s and 1990s, ‘congestive heart failure’ (CHF) was used to refer to all the HF patients. With a better understanding of pathophysiology of ‘diastolic HF’ (DHF), the term ‘HFpEF’ got widespread acceptance in early 2000s. Despite the increasing awareness of pathophysiology and diagnostic modalities for this group of HF patients, it is unfortunate to say that the therapies that we can provide are limited when compared to their counterpart HFrEF group [3].

The traditional medications that are part of GDMT for HFrEF patients were investigated in different trials including but not limited to the Perindopril in Elderly Patients with CHF (PEP-CHF) trial (angiotensin converting enzyme/ACE inhibitors), Candesartan in Heart Failure-Assessment of Reduction in Mortality and Morbidity (CHARM)-preserved trial (angiotensin receptor blockers/ARBs), the Swedish Doppler-echocardiographic study (SWEDIC) trial (beta-blockers) and the Aldosterone Receptor Blockade in DHF (Aldo-DHF) trial (aldosterone antagonist). In addition to these traditional guideline medications, use of irbesartan in the I-PRESERVE (Irbesartan in Heart Failure with Preserved Systolic Function) trial, digoxin in the DIG (Digitalis Investigation Group) trial and nevibolol in the SENIORS (Study of the Effects of Nebivolol Intervention on Outcomes and Rehospitalization in Seniors) trial was also studied. To the dismay of the investigators, none of these trials yielded positive results [4]. The only successful drug trial for this patient population is the TOPCAT (Treatment of Preserved Cardiac Function Heart Failure With an Aldosterone Antagonist) trial that demonstrated a lower hospitalization in patients with HFpEF with use of spironolactone (aldosterone antagonist). However, there was no significant difference in the primary outcome of mortality [5]. The post hoc analysis of this study by Pfeffer et al. in 2015 demonstrated greater potassium and creatinine changes and possible clinical benefits with spironolactone in patients with HFpEF from the Americas (United States, Canada, Brazil and Argentina) as compared to patients from Russia and Georgia [6]. Another recent progress is the failure of angiotensin receptor/neprilysin inhibitor sacubitril/valsartan in HFpEF patients studied in the PARAGON-HF (Prospective Comparison of ARNI with ARB Global Outcomes in HFpEF) trial in 2019. This has left the investigators with very little options to further explore. While sodium-glucose cotransporter (SLGT2) inhibitors showed improvement in primary outcomes in HFrEF patients (DAPA-HF [Dapagliflozin And Prevention of Adverse outcomes in HF] study in 2019), investigators are now trying to explore their role in HFpEF patient population [7].

Another frontier that has been studied in HFpEF patients is the use of remote hemodynamic monitoring using called CardioMEMS (Atlanta, GA), a device the remotely monitors the hemodynamics and helps the physicians to titrate diuretic treatment accordingly. It includes a delivery catheter with a hermetically sealed implantable wireless pulmonary artery (PA) sensor, hospital or patient electronic system and patient database. The PA sensor provides noninvasive hemodynamic data, which are collected in the physician’s clinic, hospital or patient’s home. The data include PA pressure waveforms, heart rate as well as systolic, diastolic and mean PA pressures. The data are transmitted to a secure website, where PA monitoring information is available at all times. Changes in PA pressure can be used in conjunction with symptoms and signs of HF to guide modifications of medical therapy. As a result of the CHAMPION (CardioMEMS Heart Sensor Allows Monitoring of Pressure to Improve Outcomes in NYHA Class III HF Patients) trial that demonstrated a lower hospitalization rate with the use of this device in HF patients irrespective of their EF, this device got approved by FDA in 2014 for New York Heart Association (NYHA class) III patients including HFpEF patients [8].

It is however unfortunate to say that, despite all these efforts, HFpEF patients continue to present the long-term management challenge especially when it is related to reducing mortality. There is a consensus in both American College of Cardiology/American Heart Association/Heart Failure Society of America (ACC/AHA/HFSA) and European society of cardiology (ESC) guidelines in the management of HFpEF patients. This basically involves aggressive modification of risk factors such as blood pressure control and diabetes management, while using diuretics to maintain the volume status [9,10].

As our knowledge of pathophysiology of HFpEF continues to enhance, it is now recognized that underlying microvascular ischemia is the key insult in these patients. Given the combined systolic and diastolic myocardial reserve limitations in HFpEF, which are demonstrable in both ventricles, it is logical to consider abnormalities in cardiomyocyte energy availability or utilization as potential contributors. Numerous recent studies suggest that coronary microvascular dysfunction may play an important role in the pathophysiology of HFpEF. Myocardial ischemia and injury are common in HFpEF and are correlated with the ventricular functional abnormalities noted in human physiological studies. Ischemia may be caused by supply demand mismatch because of high left ventricle (LV) end diastolic pressure, macrovascular (epicardial) disease and coronary microvascular dysfunction. Therefore, somewhat similar to patients with HFrEF, patients with HFpEF are also predisposed to various atrial and ventricular arrhythmias which can be fatal leading to sudden cardiac death. However, while HFrEF patients have an established indication for implantable cardioverter defibrillator (ICD) no such indication exists for HFpEF patients [11,12].

This also holds true for the use of cardiac resynchronization therapy (CRT) as well. This device improves the synchrony of contraction of left and right sides of heart to gain the maximal beneficial effect which overtime translates into improved ejection fraction (EF) and reduced mortality. Theoretically in patients with HFpEF, by improving the systolic and diastolic dyssynchrony and providing chronotropic support, this therapy can add to the diastolic filling time of the heart [13]. 

To date, data that reviewed the use of device therapy in patients with EF, which is above the current recommended guidelines (which is an EF of <35%), are very limited. One of them is a post hoc analysis of echocardiograms by Chung et al. performed in the PROSPECT trial, in which they found that 24% of patients had an LVEF above 35% and their outcome was not different compared with the group with an EF of <35%. There was a similar reduction in LV end-systolic volumes in both groups, which is a reliable end point of both morbidity and mortality [14]. The second study is a report of case by Penicka et al., who used CRT in a patient with pure DHF with left bundle branch block. Utilizing pressure-volume loops, they demonstrated a reduction in dyssynchrony after CRT. The patient also showed an improvement in functional class and in exercise capacity [15].

As diagnosis of HFpEF is based on specific echocardiographic criteria, one can argue that the patients who were analyzed by Chung et al. were not true HFpEF but borderline systolic HF or HF with intermediate EF (those with EF between 40% and 50%) patients [14]. Penicka et al. in their case described the report of one patient which does generate hypothesis but unfortunately is insufficient to make any conclusions especially with the lack of any long-term data such as six-minute walk test to demonstrate symptomatic improvement [15].

With this being said, HFpEF, however, is a disease with few effective known therapies, with a large proportion of patients in poor functional class and with a poor prognosis. There is a need to explore more frontiers to provide treatment options to this patient class, and device therapy definitely seems to be one of them [16]. While a national database like National Inpatient Sample or UK Biobank can be explored to see the outcomes of patients with HFpEF who have device therapy, this however will be limited by use of wrongly labeled International Classification of Diseases (ICD)-9/ICD-10 coding for HFpEF, and many patients may have another indications for device placement that will bias the study quite strongly. This will be in addition to other conventional biases related to observational studies. To conclude, starting with a feasibility study including patients fulfilling the diagnostic criteria for HFpEF, which then leads to a randomized controlled trial, seems to be the most appropriate option to answer this question. The challenging part will be to decide what the inclusion criteria should be. One proposed way of doing this will be to stratify the patients based on the risk factors that make them prone to fatal arrhythmias such as uncontrolled hypertension, obstructive sleep apnea, diabetes mellitus, respiratory failure (asthma or chronic obstructive pulmonary disease), pulmonary hypertension and starting with high-risk patient population first (those with more arrhythmia prone and cardiac desynchrony prone risk factors), as any effective new treatment option will be heralded even if it is only for a subgroup of patients with this devastating condition.

## Conclusions

HFpEF is a disease with few effective known therapies, with a large proportion of patients in poor functional class and with a poor prognosis. There is a need to explore more frontiers to provide treatment options to this patient class, and device therapy definitely seems to be one of them. The challenging part will be to decide what the inclusion criteria should be. There is very limited retrospective data available to predict which specific group of patients would benefit from device therapy. To test such therapy in this patient population, the subgroup which is most prone to arrhythmias and cardiac desynchrony needs to be tested as HFpEF is a broad umbrella under which multiple subgroups exist. 
